# The Correlation Between Serum Anti-tissue Transglutaminase (Anti-tTG) Antibody Levels and Histological Severity of Celiac Disease in Adolescents and Adults: A Meta-Analysis

**DOI:** 10.7759/cureus.51169

**Published:** 2023-12-27

**Authors:** Muhammad Hassan Qureshi

**Affiliations:** 1 Medicine, Combined Military Hospital, Lahore, PAK; 2 Health, Medicine, and Social Care, Anglia Ruskin University, Cambridge, GBR

**Keywords:** gluten-sensitive enteropathy, non-tropical sprue, coeliac sprue, histological severity, anti-ttg antibody, celiac disease

## Abstract

Celiac disease is an autoimmune disorder characterized by a broad spectrum of histological damage to the intestinal mucosa. Comprehension and understanding of the association between anti-tissue transglutaminase (anti-tTG) antibody levels and the histological severity of celiac disease are not well established, prompting the need for meta-analysis. This study aims to offer insights into the diagnostic abilities of anti-tTG antibody levels in determining the histological severity of celiac disease by providing quantitative evidence based on a diverse range of studies. An extensive search was conducted across four electronic research databases to identify primary research articles reporting serum anti-tTG antibody levels in correlation with different Marsh grades, signifying the histological severity of celiac disease. The software tool RevMan 5.4 (the Cochrane Collaboration, London, UK) was used to compile standardized mean differences (SMD) alongside their respective confidence intervals. A total of 13 studies were included in the meta-analysis, with a patient pool of 2505 patients. Marsh grade I and II were found to have higher anti-tTG antibody levels compared to those with grade 0 (SMD 1.50; 95% CI: 1.12, 1.87; p-value <0.00001). Antibody levels were higher in Marsh grade IIIa when compared to both grade 0 (SMD 0.97; 95% CI: 0.67, 1.28; p-value <0.00001) and grade *≤2 *(SMD 0.61; 95% CI: 0.44, 0.79; p-value <0.00001). Patients with Marsh IIIb also reported greater anti-tTG levels compared to grade 0 (SMD 1.48; 95% CI: 0.99, 1.96; p-value <0.00001) and grade *≤2 *(SMD 0.98; 95% CI: 0.79, 1.18; p-value <0.00001). Likewise, Marsh grade ≥IIIc reported high levels of anti-tTG antibodies in comparison with grade 0 (SMD 1.06; 95% CI: 0.72, 1.39; p-value <0.00001) and grade *≤2 *(SMD 1.18; 95% CI: 1.02, 1.34; p-value <0.00001). Our meta-analysis revealed a consistent, robust correlation between anti-tTG antibody levels and the histological severity of celiac disease, with a clear trend of increasing antibody levels corresponding to the severity of mucosal damage. Large-scale primary research initiatives are needed to reach definitive conclusions.

## Introduction and background

Celiac disease is an immune-mediated chronic disorder marked by the immunological response of the body to certain proteins called gluten, occurring in genetically predisposed individuals. Gluten is present in the staple diet of most cultures; anything containing wheat, rye, oats, or barley is likely to trigger a response. The classic presentation of celiac disease includes gastrointestinal manifestations such as diarrhea, bloating, abdominal discomfort, or irritability. However, it is a systemic condition that may result in injury to multiple organs upon exposure to gluten [1].

The diagnosis of celiac disease often involves a combination of clinical presentation, testing for serological markers, and a small bowel endoscopy. A duodenal biopsy is considered the ideal diagnostic test for celiac disease, given the characteristic intestinal presentation of villous atrophy, hyperplasia of the crypts, and an increase in lymphocyte infiltration of the intestinal mucosa. The limited availability, cost, and invasive nature of endoscopy enhance the importance of serological markers of celiac disease [2].

Serum anti-tissue transglutaminase (anti-tTG) antibody levels are the most frequently utilized serological test for the screening of celiac disease [2]. Other tests like anti-endomysial antibody (EMA) and anti-deamidated gliadin peptide require diagnostic precision and proficiency, besides being expensive; the anti-tTG antibody test is not only cost-effective, but it also has the highest specificity and sensitivity in diagnosing celiac disease [3]. A number of studies have reported the association of anti-tTG antibody levels with the severity of histological damage to the intestinal mucosa. The success of serological markers has decreased the need for invasive procedures such as duodenal biopsy for initial screening and diagnosis of celiac disease [4].

Current diagnostic guidelines recognize the utility of anti-tTG antibodies as part of the composite diagnostic process in celiac disease. The American College of Gastroenterology recommends the utilization of serological testing in symptomatic individuals before confirming the diagnosis with histological analysis [5]. This evolving approach reflects a shift toward non-invasive testing, recognizing the significance of serological markers, which is in complete alignment with the rationale of our study. The European Society of Pediatric Gastroenterology, Hepatology, and Nutrition (ESPGHAN) guidelines recommend establishing a diagnosis without duodenal biopsy in pediatric patients with anti-tTG levels ≥10 times the normal limit. Limited clarity is presented in the adult population [3]. Celiac disease is a disorder that can affect people of all ages, and there is a significant need for comprehensive diagnostic strategies for adult patients [6]. The precise correlation between anti-tTG antibody titers and the histological severity of celiac disease in adolescent and adult populations remains largely unexplored, and several studies report discrepant findings pointing toward the need for a meta-analysis to consolidate existing data and establish comprehensive results [7].

Comprehension and understanding of the association between anti-tTG antibody levels and the histological severity of celiac disease are of utmost importance to improve clinical decision-making. Based on our current knowledge, no existing reviews were found that discuss this correlation in adolescent and adult patients with celiac disease. This study aims to review existing literature on anti-tTG antibody levels and their correlation with histological damage to the intestinal mucosa in our population of interest. Our primary objective is to evaluate and interpret the data from the included studies to determine the magnitude and direction of the association between the two aforementioned variables. Clinical implications, including the potential diagnostic and prognostic value of anti-tTG antibody levels based on the findings of this review, will be discussed in detail. Gaps and limitations in existing literature, if any, will be emphasized to help future research prospects and improve understanding of the subject.

## Review

Methods

The correlation between serum anti-tTG antibody levels and the histological severity of celiac disease in adolescents and adults demonstrated by the meta-analysis of existing studies is the research question serving as the foundation of our meta-analysis. Our meta-analysis adhered to the standard outlined by the Preferred Reporting Items for Systematic Review and Meta-analysis (PRISMA) guidelines [8].

Data Sources and Search Strategy

PubMed, Cochrane Central, Science Direct, and Google Scholar were the four electronic research databases searched using the following keywords: "Celiac disease" OR "Coeliac disease" OR "gluten sensitive enteropathy" OR "non tropical sprue" AND "anti tissue transglutaminase" OR "anti tTG" OR "anti TTG." The only filter applied was the presence of keywords in the title, abstract, or author-specified terms; the search of databases was last visited in August 2023.

Inclusion and Exclusion Criteria

The inclusion and exclusion criteria of our meta-analysis are given in Table 1.

**Table 1:** Inclusion and exclusion criteria

	Inclusion criteria	Exclusion criteria
Studies	Original research studies including either cohort, case-control, or cross-sectional studies; both retrospective and prospective studies were included.	Review articles, case reports, editorials, diagnostic guidelines, conference abstracts, and chapters from books that could not provide required data were excluded.
Histological severity	Studies reporting histological severity of celiac disease according to or comparable to the Marsh-Oberhuber classification system.	Studies that do not report the histological severity of celiac disease.
Anti-tTG antibody levels	Mean levels of serum anti-tTG in correlation with different histological grades of celiac disease were provided or could be obtained through plots and graphs in the study.	Studies that do not report serum levels of anti-tTG antibody.
Disease of interest	Studies focusing specifically on celiac disease.	Studies based on other disease subjects that could confound the results.
Age	Studies involving participants aged 12 years and older.	Studies conducted on children <12 years of age.
Peer reviewed	Studies published in peer-reviewed journals.	Studies not published in peer-reviewed journals.
Language	Studies in the language English.	Studies not conducted in the English language.
Subjects	Studies based on human subjects.	Studies not involving human subjects.

Data Extraction

Data extraction was carried out by one reviewer using Microsoft Excel (Microsoft Corporation, Washington, USA) and relevant information was retrieved, which included study characteristics and patient demographics. Study outcomes were designed with assorted extracted anti-tTG antibody levels according to histological grading from the included studies.

Ethical Approval and Quality Assessment

Data was collected and analyzed from existing studies; ethical approval from the ethics review board of Anglia Ruskin University was obtained beforehand. Research conduct was transparent, and confidentiality protocols were adhered to throughout our study. The risk of bias assessment and quality check for each study were carried out using the adjusted Newcastle-Ottawa scale [9].

Statistical Analysis

All statistical analyses were carried out using RevMan version 5.4 (the Cochrane Collaboration, London, UK). Standardized mean difference (SMD) was used for continuous study outcomes, and a 95% confidence interval (CI) was reported in association with the findings. The I^2^ value was used to report heterogeneity among studies. The Z-score indicated a deviation of data points from the mean score, and a p-value of <0.05 was considered significant.

Results

Study Selection

**Figure 1:**
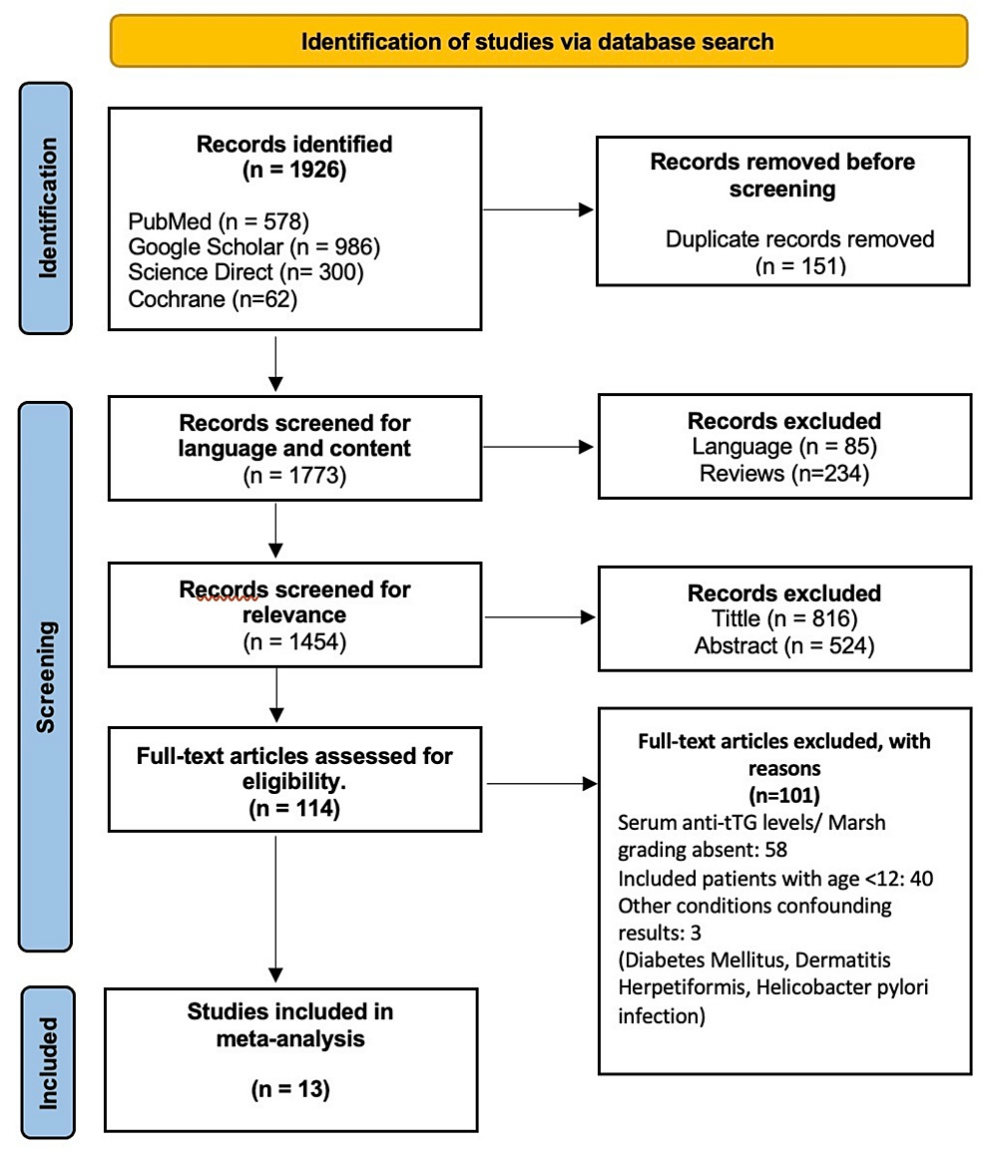
PRISMA diagram [8]

EndNote Online Classic (Clarivate, Pennsylvania, USA) was used for removing duplicate records and screening all database search results for relevance. The process of screening was carried out by one reviewer. Figure 1 shows the PRISMA diagram, which illustrates the process of study selection [8]. A total of 1926 articles were identified after the database search; 151 duplicates were removed, and the remaining 1773 entries were screened for relevance. About 1659 records were excluded from further consideration following a thorough assessment of title and abstract content; among these, 234 were review articles and 85 were not available in the English language. Access to full text was obtained for 114 articles, which were then comprehensively scrutinized according to our study criteria. Serum anti-tTG antibody levels in correlation with histological grades of celiac disease were not found in 58 articles, deeming them ineligible. Twenty-six records were based on pediatric patients, and 14 others had mixed samples of adults and children; these were also eliminated. Three articles were removed on the grounds that their study cohort was based on other medical conditions that could confound the results of our study.

Study Characteristics

Thirteen studies were included in our meta-analysis, which were in complete accordance with the inclusion criteria [10-22]. Five of the selected studies were cross-sectional, and the rest were cohort studies, divided across both prospective and retrospective study types. Studies from all over the world were included in our meta-analysis, including Hungary, India, Iran, Ireland, Italy, Romania, Sweden, and the United Kingdom. A total of 2505 patients were included in our study sample; all study characteristics are presented in detail in Table 2 [10-22]. It is significant to note that not only the units of measurement for anti-tTG antibody levels are different across most studies, but even the studies having similar measurement units have different cut-off values for anti-tTG levels. This is mainly because different methods and kits for testing anti-tTG antibody levels are applied across different studies; no universal measurement technique currently exists. On account of this reason, the result outcomes for analysis were extracted as a multiple of the upper limit of normal for each study. The unit "x ULN" represents a measurement expressed as a multiple of the upper limit of normal.

**Table 2:** Study characteristics of included studies [10-22]

Author	Journal	Study design	Country	Sample size	Age (mean ±SD)	Anti-tTG levels (mean ±SD)	Adjusted mean anti-tTG levels, x ULN
Akbari et al. [10]	European Journal of Gastroenterology and Hepatology	Cross-sectional (prospective)	Iran	29	35.45 ± 10.4	16.94 ± 29.46 AU/ml	2.42 ± 4.21 x ULN
Deane et al. [11]	Digestive and Liver Disease	Cross-sectional (retrospective)	Ireland	164	42.7 ± 12.7	8.3 x ULN	8.30 x ULN
Di Tola et al. [12]	Journal for Gastroenterology	Cross-sectional (retrospective)	-	671	34.2 ± 7.5	8.19 x ULN	8.19 x ULN
Dipper et al. [13]	Alimentary Pharmacology and Therapeutics	Cohort study (prospective)	United Kingdom	51	49.7 ± 11.9	65.9 IU/ml	4.39 x ULN
Efthymakis et al. [14]	Digestive Diseases and Sciences	Cohort study (prospective)	Italy	234	33.9 ± 11.5	14.8 ± 14.1 × ULN	14.80 ± 14.10 × ULN
Ganji et al. [15]	Middle East Journal of Digestive Diseases	Cohort study (retrospective)	Iran	299	33.0 ± 13.6	172 ± 51 IL/ml	8.60 ± 2.55 x ULN
Kalhan et al. [16]	Indian Journal of Pathology and Microbiology	Cohort study (prospective)	India	226	26.5 ± 10.3	305.28 U/ml	20.09 x ULN
Kocsis et al. [17]	European Journal of Internal Medicine	Cohort study (retrospective)	Hungary	106	38	108.25 U/ml	10.83 x ULN
Maxim et al. [18]	Revista medico-chirurgicala a Societatii de Medici si Naturalisti din Iasi	Cohort study (retrospective)	Romania	111	33 ± 12.5	122.36 U/ml	4.08 x ULN
Paul, B. et al. [19]	Annals of Pathology and Laboratory Medicine	Cohort study (prospective)	India	118	36.6 ± 15.3	10.14 U/ml	1.45 x ULN
Rathsman et al. [20]	Journal of Pathology, Microbiology and Immunology	Cross-sectional (retrospective)	Sweden	11	43.2 ± 20.4	44.5 ± 53.8 U/ml	7.42 ± 8.96 x ULN
Singh et al. [21]	Journal of Clinical Gastroenterology	Cohort study (retrospective)	India	366	28.9 ± 12.4	6.76 x ULN	6.76 x ULN
Tursi et al. [22]	Journal of Clinical Gastroenterology	Cross-sectional (prospective)	Italy	119	28 ± 7.25	30.56 ± 64 UA/ml	4.37 ± 9.14 x ULN

Risk of Bias in Included Studies

The risk of bias was carried out separately for cross-sectional and cohort studies, in accordance with the adjusted Newcastle-Ottawa scale [9]. Ten of the total 13 included studies in our meta-analysis were high-quality studies, and three studies showed some risk of bias. Table 3 illustrates the results of the bias assessment of the five cross-sectional studies [10-12,20,22]. Among these studies, Akbari et al. showed some risk of bias on the grounds of the small sample size and high rate of attrition [10].

**Table 3:** Risk of bias assessment for included cross-sectional studies [10-12,20,22]

	Selection				Comparability	Outcome		
Studies	Study clearly defines demographics of study population	Sample is representative of target population	Sample size was adequate to achieve objectives	Attrition rate was less than 20%	Study accounts for and controls potential confounding factors	Appropriate methods used to measure antibody levels and histological severity	Clear description of histological severity was provided	Statistical methods used for analyzing correlation were appropriate
Akbari et al. [10]	+	+	-	-	+/-	+	+	+
Deane et al. [11]	+	+	+	+	+/-	+	+	+
Di Tola et al. [12]	+	+	+	+	+/-	+	+	+
Rathsman et al. [20]	+	+	-	+	+/-	+	+	+
Tursi et al. [22]	+	+	+	+	+/-	+	+	+

Table 4 shows the risk of bias assessment of included eight cohort studies, and all were high-quality studies with low bias risk except two, which reported some concerns [13-19,21]. Ganji et al. (2016) neither reported attrition rate nor histological severity across the study group and also failed to account for confounding factors, resulting in a moderate risk of bias [15]. Maxim et al. (2014) showed moderate bias risk due to the absence of proper criteria of inclusion and exclusion; confounding factors were also unaccounted for, and attrition was not mentioned [18].

**Table 4:** Risk of bias assessment for included cohort studies [13-19,21]

	Selection				Comparability	Outcome		
Studies	The cohort is well-defined and adequately described	The cohort is representative of target population	Inclusion and exclusion criteria were clearly defined	Attrition rate was less than 20%?	Study accounts for and controls potential confounding factors	Assessment of anti-tTG well-defined and consistently applied	Assessment of histological severity well-defined and consistently applied	Reasonable follow-up duration present for assessing the correlation
Dipper et al. [13]	+	+	+	-	+/-	+	+	+
Efthymakis et al. [14]	+	+	+	+	+/-	+	+	+
Ganji et al. [15]	+	+	+	+/-	+/-	+	-	+
Kalhan et al. [16]	+	+	-	+	+/-	+	+	+
Kocsis et al. [17]	+	+	+	-	+/-	+	+	+
Maxim et al. [18]	+	+	-	-	+/-	+	+	+
Paul, B. et al. [19]	+	+	+	+	+/-	+	+	+
Singh et al. [21]	+	+	+	+	+/-	+	+	+

Synthesis of Results

A meta-analysis was performed to assess the statistical significance of anti-tTG antibody levels in increasing grades of histological severity according to the Marsh-Oberhuber classification when compared to Marsh grade 0 and Marsh grade ≤2 across the included studies.

Marsh I and II vs. Marsh 0

**Figure 2:**
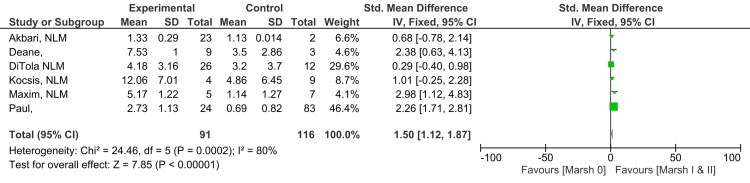
Anti-tTG antibody levels in Marsh grade I and II compared to Marsh grade 0 [10-12,17-19]

A substantial and statistically significant difference in anti-tTG antibody levels was reported between Marsh grade I and II patients in comparison to patients with an intestinal biopsy showing grade 0 (SMD 1.50; 95% CI: 1.12, 1.87). The level of heterogeneity among studies reported for this outcome was high (I^2^ = 80%). Figure 2 illustrates the result of meta-analysis across six studies that individuals with Marsh grade I and II histology have higher anti-tTG antibody levels in comparison to those with Marsh grade 0 (Z=7.85; p-value <0.00001) [10-12,17-19].

Marsh IIIa vs. Marsh 0

**Figure 3:**
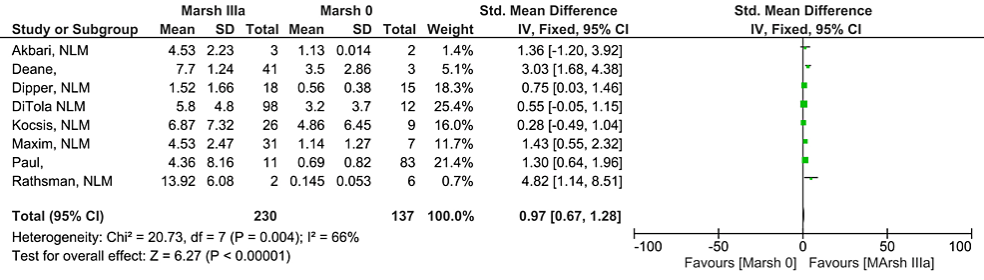
Anti-tTG antibody levels in Marsh IIIa compared to Marsh 0 [10-13,17-20]

Antibody levels in Marsh grade IIIa subjects compared to Marsh grade 0 showed a moderate to high difference, which was found statistically significant (SMD 0.97; 95% CI: 0.67, 1.28). A moderate level of heterogeneity among studies was reported for this outcome (I^2^ = 66%). Figure 3 shows results suggesting that patients with histological damage of Marsh grade IIIa have higher anti-tTG antibody levels than those with Marsh grade 0 histology (Z=6.27; p-value <0.00001) [10-13,17-20].

Marsh IIIb vs. Marsh 0

**Figure 4:**
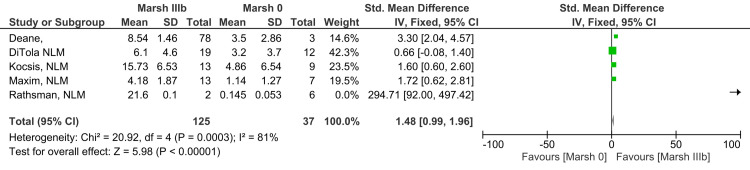
Anti-tTG antibody levels in Marsh grade IIIb compared to Marsh 0 [11,12,17,18,20]

Levels of anti-tTG antibody in subjects with grade Marsh grade IIIb compared to Marsh grade 0 revealed a highly significant difference, which means that the observed difference is not due to chance (SMD 1.48; 95% CI: 0.99, 1.96). The level of heterogeneity reported among studies for this outcome was high (I^2^ = 81%). The analysis reveals that anti-tTG antibody levels in Marsh grade IIIb are higher in comparison to Marsh grade 0, as illustrated in Figure 4 (Z=5.98; p-value <0.00001) [11,12,17,18,20].

Marsh ≥IIIc vs. Marsh 0

**Figure 5:**
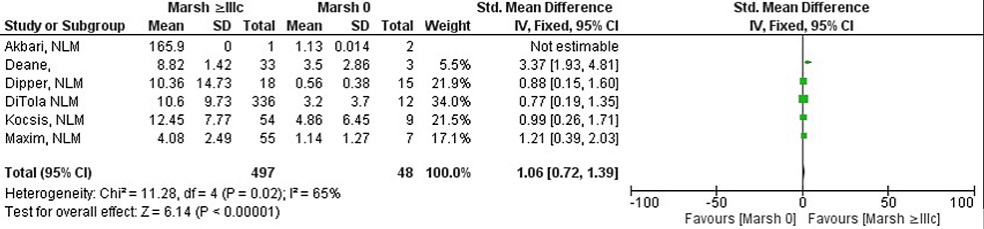
Anti-tTG antibody levels in Marsh grade ≥IIIc compared to Marsh grade 0 [10-13,17,18]

Celiac disease patients with Marsh grade ≥IIIc or higher showed a difference in anti-tTG antibody levels when compared to Marsh grade 0 patients, and the difference seen was substantial and statistically significant (SMD 1.06; 95% CI: 0.72, 1.39). The heterogeneity level among studies for this outcome was moderately high, indicating that there is some variability across studies, but it is not extreme (I^2^ = 65%). Results of the meta-analysis shown in Figure 5 indicate that anti-tTG antibody titers in patients with Marsh grade ≥IIIc are greater than antibody titers in Marsh grade 0 patients (Z=6.14; p-value <0.00001) [10-13,17,18].

Marsh IIIa vs. Marsh ≤2

**Figure 6:**
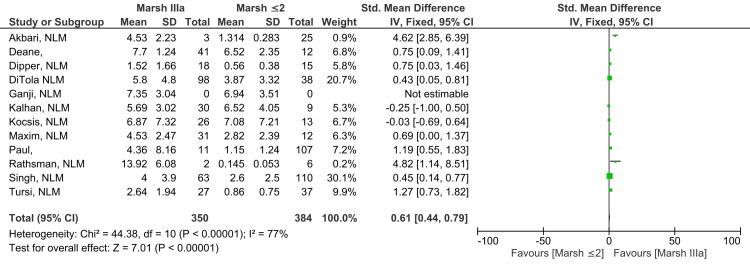
Anti-tTG antibody levels in Marsh IIIa compared to Marsh ≤2 [10-13,15-22]

A statistically significant, moderate level of difference was reported when anti-tTG antibody levels found in Marsh grade IIIa subjects were compared to levels found in subjects with Marsh grade ≤2 (SMD 0.61; 95% CI: 0.44, 0.79). The level of heterogeneity among studies was significantly high for this outcome (I^2^ = 77%). Figure 6 illustrates a meta-analysis across 12 studies, providing evidence that anti-tTG antibody levels were higher in patients with the histological grade Marsh grade IIIa in comparison to those with Marsh grade ≤2 (Z=7.01; p-value <0.00001) [10-13,15-22].

Marsh IIIb vs. Marsh ≤2

**Figure 7:**
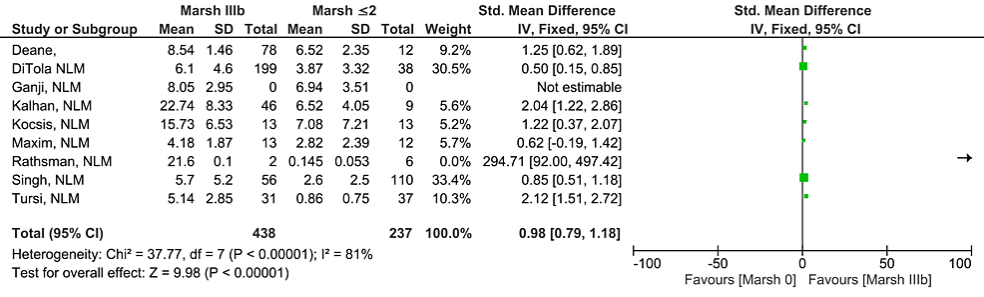
Anti-tTG antibody levels in Marsh IIIb compared to Marsh ≤2 [11,12,15-18,20-22]

Anti-tTG antibody levels in Marsh grade IIIb subjects were shown to be different from levels found in subjects showing Marsh grade ≤2, and the finding was statistically significant (SMD 0.98; 95% CI: 0.79, 1.18). There was a significantly high level of heterogeneity among the studies reported for this outcome (I^2^ = 81%). Findings illustrated in Figure 7 suggest that higher levels of anti-tTG antibody were found in Marsh grade IIIb compared to Marsh grade ≤2 (Z=9.98; p-value <0.00001) [11,12,15-18,20-22].

Marsh ≥IIIc vs. Marsh ≤2

**Figure 8:**
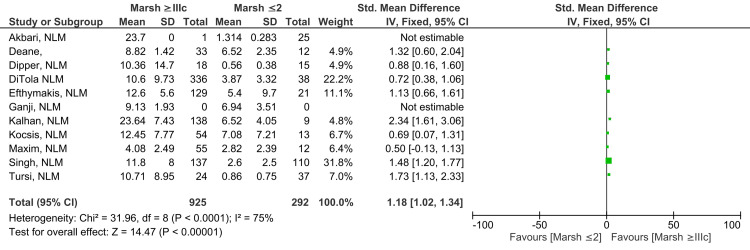
Anti-tTG antibody levels in Marsh ≥IIIc compared to Marsh ≤2 [10-18,21,22]

A statistically significant difference, likely to have clinical relevance, was reported between anti-tTG antibody levels in subjects showing Marsh grade ≥IIIc on intestinal histology compared to those with Marsh grade ≤2 (SMD 1.18; 95% CI: 1.02, 1.34). A substantially high degree of heterogeneity was observed across studies for this outcome (I^2^ = 75%). A meta-analysis comprising 11 studies illustrated in Figure 8 reveals that Marsh grade ≥IIIc showed greater anti-tTG antibody titers in contrast to those with Marsh grade ≤2 (Z=14.47; p-value <0.00001) [10-18,21,22].

Publication Bias

**Figure 9:**
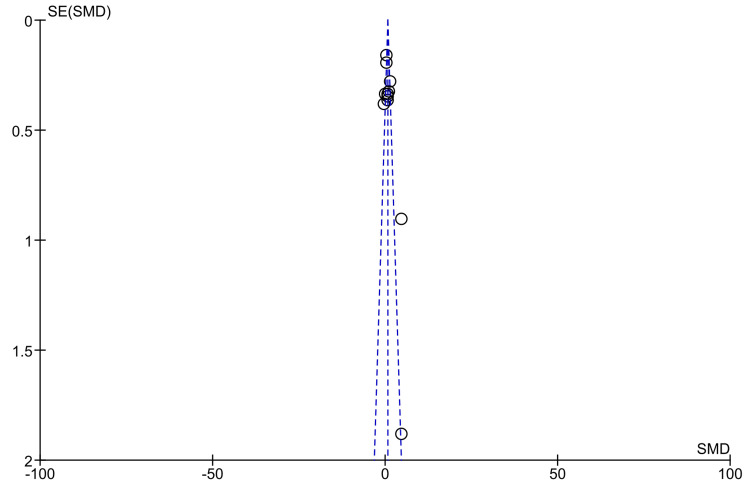
Funnel plot for anti-tTG antibody levels in Marsh IIIa compared to Marsh ≤2 [10-13,15-22]

**Figure 10:**
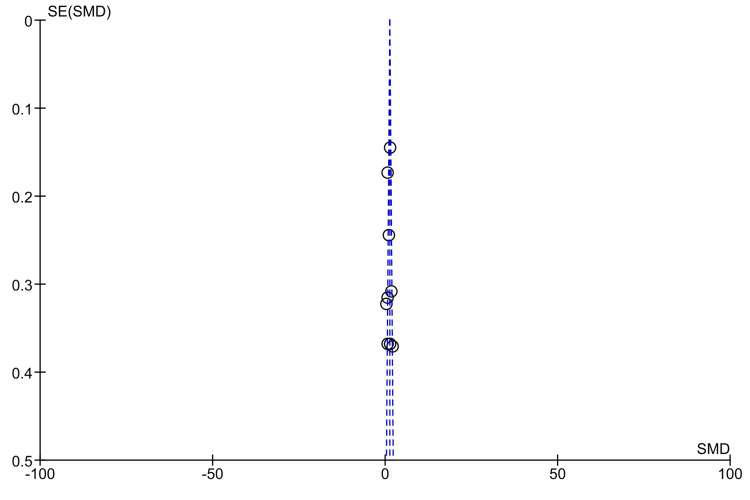
Funnel plot for anti-tTG antibody levels in Marsh ≥IIIc compared to Marsh ≤2 [10-18,21,22]

Meta-analysis was carried out for seven outcomes, and among these, only two had 10 or more studies included in the analysis. A funnel plot was created to assess publication bias for anti-tTG antibody levels found in Marsh grade IIIa and ≥IIIc in comparison to Marsh grade ≤2, as illustrated in Figure 9 and Figure 10, respectively [10-22]. The shape of the funnel plot figures revealed some degree of asymmetry, suggesting that some publication bias may exist.

Discussion

According to our current understanding, this is the first comprehensive meta-analysis that explores the relationship between anti-tTG antibody levels and the histological severity of celiac disease. All 13 studies included in our analysis collectively demonstrated the diagnostic potential of anti-tTG antibody levels and their ability to reflect the degree of damage to the intestinal mucosa. The meta-analysis methodically evaluated this correlation by comparing anti-tTG antibody levels in increasing grades of the Marsh classification with grades 0 and ≤2, separately. Detailed analyses included a comparison of Marsh grade I+II vs. 0, IIIa vs. 0, IIIb vs. 0, ≥IIIc vs. 0, IIIa vs. ≤2, IIIb vs. ≤2, and ≥IIIc vs. ≤2. Across all these comparisons, a statistically significant and consistent correlation was observed, offering crucial insights into the diagnostic potential of anti-tTG antibody levels in assessing the extent of mucosal atrophy.

Maglio et al. (2020) elaborated on the fundamental concept that anti-tTG antibodies are produced by our immune system in response to gluten exposure, ultimately contributing to damage to the intestinal mucosa. The study supports the results of our meta-analysis, arguing that anti-tTG antibodies produced in the small intestine increase during active celiac disease, resulting in intestinal damage. These findings align with the robust correlation between serum anti-tTG levels and the histological severity of celiac disease reported in our results [23].

Tonutti et al. (2014) reported findings in congruence with our meta-analysis, highlighting the significance of anti-tTG antibody levels in the diagnostic process. This qualitative review strengthens the evidence of a strong correlation between anti-tTG antibody levels and the histological severity of celiac disease, using multiple studies. Additionally, practical implications for diagnosis based on very high levels of anti-tTG antibodies are outlined in this review, reinforcing the correlation quantitatively established in our meta-analysis [24].

Sblattero et al. (2000) reported findings that align with the broader perspective presented in our meta-analysis, emphasizing the crucial role of anti-tTG antibodies in the effective diagnosis of celiac disease. In congruence with our research, this study suggests a transition from relying on biopsy results to a serology-centric diagnostic strategy. Anti-tTG antibody testing is reported to have high diagnostic potential in identifying both typical and atypical celiac disease, according to the results of this study, besides also offering the potential advantages of cost-effectiveness and early intervention. The suggested diagnostic approach by Sblattero et al. (2000) complements and validates the quantitative evidence provided by our meta-analysis to understand the relationship between anti-tTG markers and mucosal atrophy in celiac disease [25].

A comprehensive review focused on the role of anti-tTG antibodies in celiac disease discusses their key contribution to the pathogenesis of intestinal damage [26]. The autoimmune response triggered by the ingestion of gluten in celiac disease is supported by the presence of anti-tTG antibodies in the serum and intestinal mucosa. Increased uptake of tissue transglutaminase in the inflamed intestinal mucosa results in the formation of tTG-gliadin complexes by crosslinking gliadin peptides, and these complexes ultimately lead to the production of anti-tTG antibodies. The complex interplay of anti-tTG antibodies affects tTG enzymatic activity, influencing cell proliferation. This article makes use of existing literature to support the knowledge that intricate involvement of anti-tTG antibodies leads to an inflammatory response resulting in damage to the intestinal lining, reinforcing the results of our meta-analysis, which emphasizes the diagnostic potential of anti-tTG antibody levels in determining the severity of intestinal damage [26].

Another qualitative review, based on the serological marker under discussion, validates the correlation established by our meta-analysis [27]. This study complements our findings by reporting the high sensitivity and specificity of anti-tTG antibodies, in addition to elaborating on the mechanism underlying their production in celiac disease. Based on recent evidence, the review describes anti-tTG as the "master regulator" of celiac disease, showing direct involvement in the immune and cellular responses of the body to gluten exposure. This concept aligns with our results, which highlight the correlation between anti-tTG levels and histological severity, reinforcing the understanding that anti-tTG antibodies are reliable markers for assessing the degree of mucosal damage [27].

Rai et al. (2022) highlighted a crucial conundrum around significantly elevated anti-tTG antibody levels in a case series. This study followed 11 patients presenting with gastrointestinal symptoms imitating celiac disease, and anti-tTG antibody titers were carried out, which reported levels >10 x ULN. Endoscopy examination revealed giardia trophozoites, but no signs of damage to the intestinal mucosa were found. While our meta-analysis reinforces evidence supporting the role of anti-tTG antibody levels in determining intestinal damage, the findings of this study emphasize the importance of considering factors such as parasitic infestations, which may potentially confound results and yield deceptive positive findings. Essentially, this case series highlights practical challenges that may arise during the interpretation of serological findings in a clinical setting [28].

Aziz et al. (2015) stressed the complexity of the relationship between anti-tTG antibody levels and the severity of celiac disease. This study reported that while a large majority of non-celiac cases did not report raised levels of anti-tTG, almost one-third of celiac disease cases also showed normal anti-tTG levels, revealing that antibody levels can be normal in some cases despite the presence of histological damage. Moreover, this research underscores the importance of specific anti-tTG thresholds, revealing that levels >20 x ULN were seen exclusively in celiac disease in comparison to levels >2 x ULN, which showed prevalence in both celiac and non-celiac cases. When compared to the findings of our meta-analysis, the diagnostic potential of anti-tTG antibody levels in identifying intestinal damage is commonly highlighted by both studies. However, Aziz et al. (2015) elaborated on limitations such as the sensitivity and specificity of anti-tTG antibodies, highlighting the importance of additional investigations despite the presence of elevated antibody levels [29].

The diagnostic potential of anti-tTG antibody levels was recognized by our meta-analysis, affirming their ability to accurately reflect the degree of mucosal damage. There is a significant increase in anti-tTG antibody levels associated with higher Marsh grades, offering valuable insights to assess disease severity, monitor adherence to treatment, and guide effective clinical interventions. Despite the strong association between antibody levels and histological severity, our review highlights the intricate nature of celiac disease diagnosis in certain cases. This generally robust correlation may be complicated by decreased antibody titers in cases of immunoglobulin deficiency or early mucosal damage. Similarly, antibody levels may be falsely raised in certain conditions, such as giardiasis or autoimmune diseases. Conflicting serological and histological results may often arise, stressing the importance of a comprehensive diagnostic approach and clinical judgment.

A major strength of our meta-analysis is the presence of broad inclusion criteria encompassing a wide range of studies, leading to a comprehensive understanding of the topic. The included studies underwent thorough assessment for quality and publication bias, and all the studies were identified as high-quality studies. Primary research articles included in our meta-analysis were conducted in various countries, and this diversity provides the foundation for generalization and increases the credibility of our research outcomes.

It is crucial to acknowledge and consider the limitations of our meta-analysis. The reliability of one reviewer for the search and selection of studies significantly increased the chances of our study being subject to selection bias, resulting in a considerable risk of error and subjectivity. A single reviewer conducted both the extraction and analysis of the data, introducing potential bias elements. In some studies, mean anti-tTG antibody levels in correlation to different histological grades were extracted from graphs with large-scale calibrations that might have caused a decrease in inaccuracy. Out of the 13 included studies, seven were retrospective; this design of the study may introduce potential recall bias, data collection problems, and limited control over confounding variables.

Another inherent limitation of our review lies in the utilization of study-level meta-analysis, which compiles data from a total number of subjects included in studies instead of results for individual patients or trials. This fails to consider the diversity in demographic variables and clinical presentation across individuals. A high degree of heterogeneity existed in our meta-analysis, largely due to the absence of a universally adopted method for antibody measurement and variations in cut-off values. The limitation of a smaller sample size of included studies might also limit the statistical power of our analysis.

The need for studies of large sample sizes evaluating the correlation between antibody levels and the severity of intestinal mucosal damage must be addressed. The conduct of rigorous studies encompassing diverse patient populations, including different geographical locations, ethnicities, and disease severity, is required to thoroughly study clinical significance. Further primary research and trials are essential to secure and strengthen conclusive results about the diagnostic potential of anti-tTG antibody levels. Future research should also focus on the standardization of assessment methods for anti-tTG antibodies and defining reference values across different Marsh grades. Longitudinal studies assessing changes in anti-tTG levels over the period of disease progression and their correlation with clinical symptoms may help in a comprehensive understanding of the disease.

## Conclusions

The meta-analysis comprehensively explored the correlation between anti-tTG antibody levels and the histological severity of celiac disease across diverse populations and research methods. The amalgamation of results presented a positive correlation between increased anti-tTG levels and histological severity, showing a clear trend of increasing anti-tTG levels associated with higher Marsh grades, implying more severe lesions.

Our meta-analysis provides quantitative evidence, offering valuable insights into the relationship between serum anti-tTG antibody levels and the histological severity of celiac disease. This consistent, strong association validates the key role of anti-tTG antibody levels in the diagnosis and management of celiac disease, providing guidance to clinicians and researchers. The significance of these findings underscores the need for extensive randomized control trials to establish novel diagnostic guidelines. The highlighted feasibility of such trials ensures a valuable contribution to diagnostic strategies by validating the effectiveness of anti-tTG antibody levels in the diagnosis and staging of celiac disease.
